# Supplementary material for: Directed Recovery and Molecular Characterization of Antibiotic Resistance Plasmids from Cheese Bacteria
Source: Int J Mol Sci. 2021 Jul 21;22(15):7801. doi: 10.3390/ijms22157801 (PMC8346141; doi:10.3390/ijms22157801)
Supplement: Supplementary file 1 [file ijms-22-07801-s001.zip › ijms-1275647-supplementary.pdf]

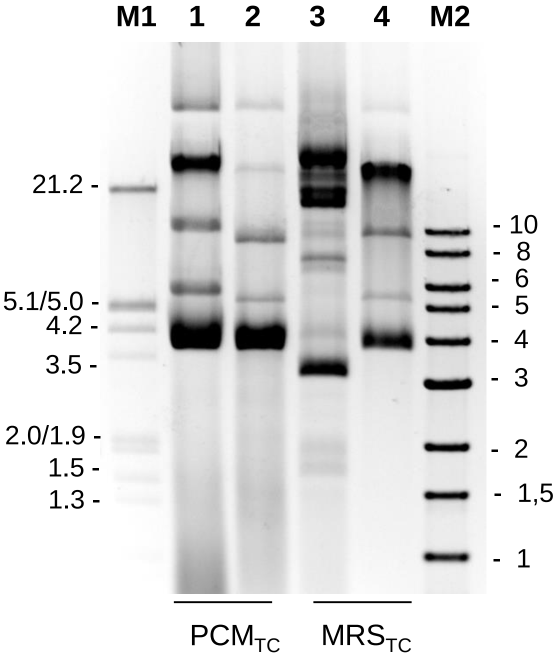

Figure S1.- Gel electrophoresis of total plasmid DNA from bacteria recovered in bulk from Plate Count Milk (PCM) and de Man Rogosa and Sharpe (MRS) agar plates supplemented with tetracycline (25 mg mL^{-1}) and digested with PstI and XhoI. Lanes: 1, PCM_{TC}-3D; 2, PCM_{TC}-60D; 3, MRS_{TC}-3D; and 4, MRS_{TC}-60D. M1, lambda DNA digested with EcoRI and HindIII (kbp), and M2, GRS universal ladder (kbp; GRiSP).

Table S1.- Open reading frame (ORF) analysis of tetracycline (pTC1 through pTC7) and erythromycin (pERM1) resistance plasmids of this study recovered after transformation of total plasmid DNA from cheese bacteria into *Lactococcus lactis* NZ9000, *Lacticaseibacillus casei* BL23, and *Lactiplantibacillus plantarum* NC8. Color code of the ORFs: in yellow, genes encoding replication and plasmid maintenance-associated proteins; in green, genes encoding integrases, recombinases, and mobilization-involved proteins; in red, antibiotic resistance genes.

ORFs	5' end position	3' end position[a]	% GC content[b]	No. of aa[c]	Known protein with the highest homology, microorganism/microbial group	aa[b] length/total length (% aa identity)	GenBank accession no.
**pTC1 from *Lactococcus lactis* NZ9000**							
ORF1	340	11,037	37.2	325	LPXTG cell wall anchor domain-containing protein, *Streptococcus parauberis*	325/325 (100%)	WP_100190763.1
ΔORF2	501	650	27.3	49	Conjugal transfer protein, Multispecies	49/67 (100%)	WP_017370349.1
ORF3	1,479	3,419	33.3	646	Tetracycline resistance ribosomal protection protein Tet(S), *Enterococcus faecium*	646/646 (100%)	WP_002405437.1
ORF4	3,693	3,514	37.2	56	Hypothetical protein/GNAT family N-acetyltransferase, *S. parauberis*	55/59 (100%)	EMG24350.1
ORF5	4,256	3,669	33.5	195	DUF536 domain-containing protein/RepB-like protein, Lactobacillales	195/195 (100%)	WP_003109091.1
ORF6	5,574	4,249	36.9	441	RepB family plasmid replication initiator protein, Lactobacillales	441/441(100%)	WP_003109089.1
ORF7	5,643	5,819	28.2	58	Hypothetical protein		
ORF8	6,572	5,970	32.5	200	Cell division protein Fic (MobC2), Lactobacillales	198/200 (99%)	WP_002395946.1
ORF9	7,212	6,589	37.3	207	Mobilization protein, Lactobacillales	206/207 (99%)	WP_063282541.1
ORF10	8,441	7,209	40.7	410	Relaxase/mobilization nuclease domain-containing protein, *Lactococcus piscium*	408/410 (99%)	WP_097024212.1
ORF11	8,914	8,420	34.7	164	Bacterial mobilization protein MobC, *Enterococcus faecalis*	164/164 (100%)	EFT90172.1
ORF12	9,014	9,301	34.7	95	Hypothetical protein, *Bacillus licheniformis*	94/95 (99%)	KUL06623.1
ORF13	9,877	9,413	35.9	154	Integrase/recombinase plasmid associated, *Lactococcus lactis* subsp. *cremoris*	154/154 (100%)	ARE24744.1
ORF14	10,202	9,906	24.9	98	DUF4298 domain-containing protein, Bacilli	98/98 (100%)	WP_003331352.1
ORF15	10,576	10,409	26.7	55	Hypothetical protein		
ORF16	10,685	10,563	31.70	40	Hypothetical protein, *L. lactis* subsp. *lactis*	40/40(100%)	ADZ65061.1
**pTC2 from *Lactococcus lactis* NZ9000**							
ORF1	565	10,557	36.24	400	BspA family leucine-rich repeat surface protein, *Streptococcus parauberis*	400/400 (100%)	WP_081247919.1
ΔORF2	651	800	27.33	49	Conjugal transfer protein, Multispecies	49/67 (100%)	WP_017370349.1
ORF3	1,629	3,569	33.33	646	Tetracycline resistance ribosomal protection protein Tet(S), *Enterococcus faecium*	646/646 (100%)	WP_002405437.1
ORF4	3,831	3,664	37.22	56	Hypothetical protein/GNAT family N-acetyltransferase, *S. parauberis*	55/59 (100%)	EMG24350.1
ORF5	4,406	3,819	33.50	195	DUF536 domain-containing protein/RepB-like protein, Lactobacillales	195/195 (100%)	WP_003109091.1

ORF6	5,724	4,399	36.95	441	RepB family plasmid replication initiator protein, Lactobacillales	441/441(100%)	WP_003109089.1
ORF7	5,793	5,966	28.24	58	Hypothetical protein		
ORF8	6,722	6,120	32.50	200	Cell division protein Fic (MobC2), Lactobacillales	198/200 (99%)	WP_002395946.1
ORF9	7,362	6,739	37.33	207	Mobilization protein, Lactobacillales	206/207 (99%)	WP_063282541.1
ORF10	8,591	7,359	40.79	410	Relaxase/mobilization nuclease domain-containing protein, *Lactococcus piscium*	408/410 (99%)	WP_097024212.1
ORF11	9,064	8,570	34.74	164	Bacterial mobilization protein MobC, *Enterococcus faecalis*	164/164 (100%)	EFT90172.1
ORF12	9,171	9,374	33.81	68	Hypothetical protein, *Lactococcus lactis*	58/94 (89%)	WP_101913476.1
ORF13	10,202	9,633	35.43	189	Site-specific recombinase, *L. lactis* subsp. *cremoris*	189/189 (100%)	AFK83769.1
ORF14	10,370	10,224	25.17	48	Hypothetical protein		
**pTC3 from *Lactococcus lactis* NZ9000**							
ORF1	273	854	26.2	193	Hypothetical protein, *Lactococcus lactis*	192/193 (99%)	WP_012898732.1
ORF2	940	1,200	33.7	86	Hypothetical protein, *L. lactis*	86/86 (100%)	WP_010891647.1
ORF3	1,294	2,205	42.8	303	Magnesium transporter CorA family protein, *L. lactis*	303/303 (100%)	WP_010891392.1
ORF4	2,375	2,537	36.4	53	Putative integrase/recombinase plasmid associated, *L. lactis*	53/53 (100%)	WP_002411279.1
ORF5	2,664	2,987	34.2	107	Cytochrome B, Lactobacillales	103/107 (96%)	WP_167545038.1
ORF6	3,653	3,931	26.1	92	Hypothetical protein, *L. lactis* subsp. *lactis*	79/92 (86%)	ARE02361.1
ORF7	4,009	5,169	34.9	386	RepB family plasmid replication initiator protein, *L. lactis*	385/386 (99%)	WP_138406205.1
ORF8	5,162	5,788	31.5	208	Replication-associated protein/DUF536 domain-containing protein, *L. lactis* subsp. *cremoris*	183/208 (100%)	WP_015062857.1
ORF9	5,797	6,027	33.3	76	Replication-associated protein RepC, *L. lactis*	76/76 (100%)	WP_010868915.1
ORF10	6,481	6,604	30.0	40	Hypothetical protein, *L. lactis* subsp. *lactis* biovar. *diacetylactis*	37/40 (93%)	AVR65363.1
ΔORF11	6,684	7,079	26.5	131	Mobilization protein MobC, *L. lactis* subsp. *lactis* biovar. *diacetylactis*	127/160 (97%)	QEX47727.1
ORF12	7,815	8,993	33.6	392	RepB family plasmid replication initiator protein, *L. lactis*	392/392 (100%)	WP_046781145.1
ORF13	8,990	9,631	31.3	213	DUF536 domain-containing protein, *L. lactis*	213/223 (96%)	WP_143460173.1
ORF14	9,640	10,140	35.5	166	Hypothetical protein, *L. lactis* subsp. *lactis*	166/166 (100%)	KHE75756.1
ORF15	10,145	10,468	29.9	107	Hypothetical protein, *L. lactis*	106/107 (99%)	WP_010890673.1
ORF16	11,772	11,086	37.5	228	IS6-like element IS*1216* family transposase, Bacteria	227/228 (99%)	WP_002354485.1
ORF17	11,850	12,054	27.9	67	Conjugal transfer protein, Multispecies	67/67 (100%)	WP_017370349.1
ORF18	12,882	14,822	33.3	646	Tetracycline resistance ribosomal protection protein Tet(S), *Enterococcus faecium*	646/646 (100%)	ABB97394.1
ORF19	14,910	15,078	35.1	55	Conjugal transfer protein, Bacilli	54/55 (98%)	WP_005228365.1
ORF20	15,208	15,097	29.8	37	Hypothetical protein, *Enterococcus faecium*	37/37 (100%)	ADA62734.1
ORF21	15,950	15,592	36.1	119	Helix-turn-helix domain-containing protein/Tn*916* transcriptional regulator, Bacilli	119/119 (100%)	WP_002405438.1

ORF22	16,206	16,475	30.0	89	Hypothetical protein, *E. faecalis*	88/89 (99%)	AEF32547.1
ORF23	16,426	16,656	37.7	76	Hypothetical protein, *L. lactis*	76/76 (100%)	WP_011997529.1
ORF24	17,394	16,708	37.4	228	IS6-like element, IS*1216* family transposase, Firmicutes	227/228 (99%)	WP_002377886.1
ΔORF25	17,587	17,897	26.0	103	Replication initiation protein, partial. *E. faecium*	103/103 (100%)	ELB42280.1
ORF26	18,033	18,680	24.0	215	Type A-8 chloramphenicol *O*-acetyltransferase, Lactobacillales	215/215 (100%)	WP_002403315.1
ORF27	18,932	20,144	35.0	403	Plasmid recombination enzyme, Lactobacillales	403/403 (100%)	WP_002328823.1
ΔORF28	20,480	20,313	46.7	71	IS*3* family transposase, *Enterococcus*	64/255 (90%)	WP_096541189.1
ORF29	20,687	20,831	39.5	47	Hypothetical protein, *E. faecalis*	46/47 (98%)	EOF23773.1
ΔORF30	20,842	21,682	31.1	279	Replication initiation protein, *Staphylococcus*	278/3147 (99%)	WP_071561775.1
ORF31	21,689	22,537	24.7	282	Streptomycin adenylyltransferase Str, Bacteria	282/282 (100%)	WP_010711054.1
ORF32	23,442	22,756	37.2	228	IS6-like element, IS*1216* family transposase, Firmicutes	227/228 (99%)	WP_002377886.1
ΔORF33	23,496	23,774	36.9	92	Type I restriction-modification system, HsdM, *L. lactis* subsp. *cremoris*	86/184 (95%)	
ORF34	23,758	200	33.3	408	Type I restriction-modification system, HsdS, *Lactococcus raffinolactis*	312/412 (75%)	WP_096040584.1
**pTC4 from *Lactococcus lactis* NZ9000**							
ORF1	340	41831	30.6	275	Hypothetical protein, *Lactococcus lactis*	275/275 (100%)	WP_010890676.1
ORF2	681	337	31.8	114	PadR family transcriptional regulator, *L. lactis*	113/114 (99%)	WP_010890649.1
ΔORF3	985	1,443	38.9	152	IS6-like element IS*Teha2* family transposase, partial, Lactobacillales	152/226 (100%)	WP_003106709.1
ORF4	1,743	1,934	32.2	63	Hypothetical protein		
ORF5	2,118	2,306	31.2	61	Putative role in replication, *L. lactis* subsp. *lactis*	61/62 (100%)	KSU18016.1
ORF6	2,462	2,286	30.5	58	Hypothetical protein		
ORF7	2,556	2,693	36.2	45	Lactococcin family bacteriocin, *Lactococcus*	30/45 (68%)	WP_081042642.1
ORF8	2,706	3,417	25.7	235	CPBP family intramembrane metallo-endoprotease, *Lactococcus*	186/236 (79%)	WP_058208202.1
ORF9	5,584	3,542	30.2	680	MobA/MobL family protein, *L. lactis*	676/680 (99%)	WP_010890626.1
ORF10	5,853	6,137	21.4	94	Hypothetical protein, *L. lactis*	94/94 (100%)	WP_052206610.1
ORF11	6,150	6,416	19.4	88	Hypothetical protein, *L. lactis*	88/88 (100%)	WP_058218801.1
ORF12	6,905	6,705	18.9	66	Hypothetical protein, *L. lactis*	65/66 (98%)	WP_058147838.1
ORF13	7,281	6,919	29.7	120	Hypothetical protein, *L. lactis*	101/118 (95%)	WP_010890683.1
ORF14	7,496	7,347	37.3	49	Hypothetical protein		
ORF15	8,883	7,714	34.1	389	RepB family plasmid replication initiator protein, *L. lactis*	389/389 (100%)	WP_058218799.1
ORF16	10,499	9,555	28.8	314	Toprim domain-containing protein, *L. lactis*	314/314 (100%)	WP_011997519.1
ORF17	10,905	10,525	30.4	126	Single-stranded DNA-binding protein, *Lactococcus*	126/126 (100%)	WP_010890681.1
ORF18	11,285	10,920	28.6	121	Hypothetical protein, *Lactococcus*	121/121 (100%)	WP_011997518.1
ORF19	11,652	11,353	27.6	99	Hypothetical protein, *Lactococcus*	99/99 (100%)	WP_011997517.1
ORF20	12,422	11,655	27.8	255	ParA family protein, *L. lactis*	255/255 (100%)	WP_011997516.1
ORF21	13,082	12,510	28.9	190	Recombinase family protein, *L. hircilactis*	190/190 (100%)	WP_153497015.1

ORF22	13,281	13,523	27.1	80	Helix-turn-helix transcriptional regulator, Xre family, *L. lactis*	80/80 (100%)	WP_101944591.1
ORF23	13,510	13,917	33.0	135	ATP-dependent helicase, *Listeria monocytogenes*	116/128 (96%)	EAD8123299.1
ORF24	14,244	14,513	30.0	89	Hypothetical protein, *Enterococcus faecalis*	88/89 (99%)	AEF32547.1
ORF25	14,515	14,694	36.6	59	DNA-binding protein, *L. lactis* subsp. *lactis*	59/59 (100%)	ARE12296.1
ORF26	15,434	14,748	37.7	228	IS6-like element, IS*1216* family transposase, Bacilli	228/228 (100%)	WP_031942290.1
ΔORF27	15,512	15,715	27.9	67	Conjugal transfer protein, *Lactococcus*	67/81 (100%)	WP_012358612.1
ORF28	16,544	18,484	33.3	646	Tetracycline resistance ribosomal protection protein Tet(S), *Enterococcus faecium*	646/646 (100%)	ABB97394.1
ORF29	19,612	19,253	36.1	119	Helix-turn-helix domain-containing protein/Tn*916*, transcriptional regulator, Bacilli	119/119 (100%)	WP_002405438.1
ORF30	19,869	20,138	30.0	89	Hypothetical protein, *E. faecalis*	88/89 (99%)	AEF32547.1
ΔORF31	20,140	20,319	36.6	59	Sigma-70 family RNA polymerase sigma factor, Lactobacillales	59/140 (100%)	WP_010774560.1
ΔORF32	20,897	20,373	37.5	174	IS6-like element, IS*1216* family transposase, partial, *Enterococcus faecium*	168/170 (99%)	WP_085842503.1
ΔORF33	21,048	20,854	31.7	64	IS6-like element, IS*1216* family transposase, partial, *L. lactis*	58/239 (97%)	WP_060416618.1
ORF34	21,539	22,450	28.1	303	MarR family transcriptional regulator, *Lactococcus*	302/303 (99%)	WP_060416617.1
ORF35	22,960	23,511	35.6	183	Recombinase family protein, *L. lactis*	181/183 (99%)	WP_046780955.1
ORF36	24,916	23,651	29.1	421	Hypothetical protein/ABC permease, *L. lactis*	421/421 (100%)	KEY61407.1
ORF37	25,808	24,924	32.0	294	ABC transporter ATP-binding protein, *L. lactis*	293/294 (99%)	WP_042748911.1
ORF38	26,176	25,811	32.7	121	GntR family transcriptional regulator, *L. lactis*	121/121 (100%)	WP_017864521.1
ORF39	28,539	26,581	36.8	652	CAP domain-containing protein, *L. lactis*	635/652 (97%)	WP_134798152.1
ORF40	28,734	29,768	37.2	344	C39 family peptidase, *L. lactis*	331/344 (100%)	WP_046781133.1
ORF41	29,792	31,012	36.7	406	Hypothetical protein, *L. lactis* subsp. *cremoris*	405/406 (99%)	KKW74469.1
ORF42	31,763	31,083	38.4	226	IS6 family transposase, *L. lactis*	218/226 (96%)	WP_081196212.1
ΔORF43	32,292	32,495	39.2	67	RepB family plasmid replication initiator protein, partial, *L. lactis*	65/94 (97%)	WP_032946768.1
ORF44	32,492	33,206	33.0	237	Hypothetical protein, *L. lactis*	222/237(94%)	WP_063284114.1
ΔORF45	33,505	33,834	29.0	109	C40 family peptidase, *L. lactis*	103/320 (100%)	WP_052750609.1
ΔORF46	33,827	34,621	37.6	264	C40 family peptidase, *L. lactis*	264/371(100%)	WP_015063492.1
ORF47	34,635	35,156	35.8	173	Hypothetical protein, *L. lactis*	172/173 (99%)	WP_015063491.1
ORF48	36,113	35,427	37.4	228	IS6-like element, IS*1216* family transposase, *E. faecium*	227/228 (99%)	WP_013558432.1
ORF49	36,862	36,641	35.1	73	Hypothetical protein, *L. lactis*	72/73 (99%)	WP_167593811.1
ORF50	37,193	36,897	32.9	98	Hypothetical protein, *Lactococcus*	98/98 (100%)	WP_003132842.1
ORF51	37,481	37,729	37.7	82	Lactococcin family bacteriocin, *L. lactis*	82/82 (100%)	WP_081041441.1
ORF52	37,731	38,012	30.1	93	Hypothetical protein, *L. lactis*	92/93 (99%)	WP_011117207.1
ΔORF53	38,707	38,198	27.8	170	CPBP family intramembrane metallo-endoprotease, *L. lactis*	169/219 (99%)	WP_021215271.1
ORF54	39,261	38,809	27.5	150	Hypothetical protein, *L. lactis*	149/150(99%)	WP_058224044.1
ORF55	40,243	39,563	38.7	226	IS6 family transposase, *L. lactis*	225/226 (99%)	WP_096816700.1

ORF56	40,400	40,549	34.0	49	Lactococcin family bacteriocin, *Lactococcus*	49/49 (100%)	WP_017865277.1
ΔORF57	40,735	41,364	29.0	209	CPBP family intramembrane metalloprotease, *L. lactis*	209/234 (100%)	WP_017865276.1
ΔORF58	41,694	41,428	38.5	88	Transposase of IS*946V*, partial, *L. lactis* subsp. *lactis*	79/167 (99%)	SBW31913.1
**pTC5 from *Lactococcus lactis* NZ9000**							
ORF1	340	31003	30.7	275	Hypothetical protein, *Lactococcus lactis*	275/275 (100%)	WP_010890676.1
ORF2	681	337	31.8	114	PadR family transcriptional regulator, *L. lactis*	113/114 (99%)	WP_010890649.1
ΔORF3	985	1,443	38.9	152	IS6-like element IS*Teha2* family transposase, Lactobacillae	152/226 (100%)	WP_003106709.1
ORF4	1,466	1,624	23.8	52	Hypothetical protein		
ORF5	2,507	2,286	27.4	73	Hypothetical protein		
ORF6	2,556	2,693	36.2	45	Lactococcin family bacteriocin, *L. lactis*	30/45 (68%)	WP_021214958.1
ORF7	2,706	3,416	25.7	236	CPBP family intramembrane metalloprotease, *Lactococcus*	186/236 (79%)	WP_058208202.1
ORF8	5,584	3,542	30.2	680	MobA/MobL family protein, *L. lactis*	676/680 (99%)	WP_010890626.1
ORF9	5,853	6,137	21.4	94	Hypothetical protein, *L. lactis*	94/94 (100%)	WP_052206610.1
ORF10	6,150	6,416	19.4	88	Hypothetical protein, *L. lactis*	88/88 (100%)	WP_058218801.1
ORF11	6,905	6,705	18.9	66	Hypothetical protein, *L. lactis*	65/66 (98%)	WP_058147838.1
ORF12	7,236	6,918	31.7	105	Hypothetical protein, *Lactococcus petauri*	100/105(95%)	
ORF13	7,496	7,347	37.3	49	Hypothetical protein		
ORF14	8,883	7,714	34.1	389	RepB family plasmid replication initiator protein, *L. lactis*	389/389 (100%)	WP_058218799.1
ORF15	10,499	9,555	28.8	314	Toprim domain-containing (ribonuclease-like) protein, *L. lactis*	314/314 (100%)	WP_011997519.1
ORF16	10,905	10,525	30.4	126	Single-stranded DNA-binding protein, *Lactococcus*	126/126 (100%)	WP_010890681.1
ORF17	11,285	10,920	28.6	121	Hypothetical protein, *Lactococcus*	121/121 (100%)	WP_011997518.1
ORF18	11,652	11,353	27.6	99	Hypothetical protein, *Lactococcus*	99/99 (100%)	WP_011997517.1
ORF19	12,422	11,655	27.8	255	ParA family protein, *L. lactis*	255/255 (100%)	WP_011997516.1
ORF20	13,082	12,510	28.9	190	Recombinase family protein, *Lactococcus hircilactis*	190/190 (100%)	WP_153497015.1
ORF21	13,281	13,523	27.1	80	Helix-turn-helix transcriptional regulator, *L. lactis*	80/80 (100%)	WP_101944591.1
ORF22	13,510	13,917	33.0	135	ATP-dependent helicase, *Listeria monocytogenes*	116/128 (96%)	EAD8123299.1
ORF23	14,082	14,234	30.7	50	Hypothetical protein		
ORF24	14,244	14,513	30.0	89	Hypothetical protein, *Enterococcus faecalis*	88/89 (99%)	AEF32547.1
ORF25	14,515	14,694	36.6	59	Hypothetical protein, *L. lactis*	58/59 (98%)	ARE12296.1
ORF26	15,434	14,748	37.4	228	IS6-like element IS*1216* family transposase, Bacteria	228/228 (100%)	WP_002354485.1
ΔORF27	15,499	15,786	32.9	95	Replication protein RepB, partial, *Enterococcus faecium*	95/125 (100%)	WP_002328826.1
ORF28	16,243	16,401	27.6	52	Hypothetical protein		
ORF29	16,633	16,454	33.8	59	Hypothetical protein		
ORF30	16,916	17,863	32.1	315	Replication initiation factor domain-containing protein, *Staphylococcus aureus*	293/314 (93%)	WP_031915289.1
ORF31	17,871	18,719	24.7	282	Streptomycin adenylyltransferase Str, Bacteria	282/282 (100%)	WP_010711054.1

ORF32	19,026	19,310	27.3	94	Replication initiation protein, *Enterococcus*	94/94 (100%)	WP_002328825.1
ORF33	19,445	20,092	24.0	215	Type A-8 chloramphenicol *O*-acetyltransferase, Lactobacillales	215/215 (100%)	WP_002403315.1
ORF34	20,345	21,556	34.8	403	Plasmid recombination protein, Lactobacillales	403/403 (100%)	WP_002328823.1
ORF35	22,100	22,255	29.4	51	Protein RepA, partial, Bacteria	50/51 (98%)	WP_002328822.1
ΔORF36	22,379	22,762	34.7	121	Replication protein RepB, *Enterococcus*	121/181 (100%)	WP_100970614.1
ORF37	23,482	22,796	37.4	228	IS6-like element, IS*1216* family transposase, Bacteria	228/228 (100%)	WP_002354485.1
ΔORF38	23,560	23,763	27.9	67	Conjugal transfer protein, Lactobacillales	67/305 (100%)	WP_002405436.1
ORF39	24,592	26,532	33.3	646	Tetracycline resistance ribosomal protection protein Tet(S), *Enterococcus faecium*	646/646 (100%)	ABB97394.1
ORF40	27,657	27,301	36.1	119	Helix-turn-helix domain-containing protein/Tn*916*, transcriptional regulator, Bacilli	118/119 (100%)	WP_002405438.1
ORF41	27,755	27,907	30.7	50	Hypothetical protein	88/89 (99%)	
ORF42	27,917	28,186	30.0	89	Hypothetical protein, *E. faecalis*	88/89 (99%)	AEF32547.1
ORF43	28,188	28,367	36.6	59	Hypothetical protein, *L. lactis*	58/59 (98%)	ARE12296.1
ORF44	29,140	28,421	36.8	239	IS6-like element, IS*1216* family transposase, *L. lactis*	239/239 (100%)	WP_060416618.1
ORF45	29,632	30,542	28.1	303	MarR family transcriptional regulator, *Lactococcus*	302/303 (99%)	WP_060416617.1
ORF46	31,010	30,843	23.2	55	Hypothetical protein		
**pTC6.1 from *Lacticaseibacillus casei* LB23**							
ORF1	999	1,856	38.3	285	Replication protein, *Lactiplantibacillus plantarum*	285/319 (100%)	WP_011222005.1
**pTC6.2 from *Lacticaseibacillus casei* LB23**							
ORF1	168	329	51.8	53	Hypothetical protein, *Schleiferilactobacillus perolens*	53/53 (100%)	KRL07062.1
ORF2	580	738	30.2	52	Ribbon-helix-helix protein (RepC), CopG family, Bacilli	52/52 (100%)	WP_011222011.1
ORF3	805	1,464	37.8	219	Replication protein (RepB), *Lactiplantibacillus plantarum*	219/219 (100%)	WP_011222008.1
ORF4	2,066	1,656	27.4	136	Hypothetical protein, *L. plantarum*	136/148 (100%)	WP_011222009.1
**pTC7 from *Lactiplantibacillus plantarum* NC8**							
ORF1	246	100	34.0	48	ABC transporter ATP-binding protein, *Lactiplantibacillus plantarum*	48/48(98%)	PHV52299.1
ORF2	345	1,046	42.4	233	Transposase- IS*30*-like element IS*Lpl1*, *L. plantarum*	232/233 (99%)	AAM61874.1
ORF3	1,224	1,670	37.4	125	Hypothetical protein, *L. plantarum*	125/125 (99%)	MBA2820843.1
ORF4	1,793	1,993	40.3	66	Cold-shock protein, Bacilli	66/66 (100%)	WP_003643340.1
ORF5	2,080	2,322	37.9	80	Hypothetical protein, *Lactobacillaceae*	80/80 (100%)	WP_003643341.1
ORF6	2,930	3,490	38.5	186	DUF536 domain-containing protein, Bacilli	186/186 (100%)	WP_003643342.1
ORF7	3,492	3,671	31.1	59	Hypothetical protein, *Lactiplantibacillus pentosus*	59/59 (100%)	RMW46525.1
ORF8	4,517	4,705	32.8	62	Hypothetical protein		

ORF9	4,822	4,670	34.0	51	Hypothetical protein, *L. plantarum* subsp. *plantarum*	51/59 (100%)	EFK30800.1
ORF10	6,692	5,898	36.7	264	Tyrosine recombinase/integrase, *L. plantarum* subsp. *plantarum*	264/264 (100%)	KRL32889.1
ORF11	6,490	6,834	34.2	114	PemI, *Weissella cibaria*	114/114 (99%)	AUG89724.1
ORF12	6,828	7,172	36.2	114	PemK, *L. plantarum*	114/114 (100%)	WP_071543014.1
**pEMR1 from *Lactococcus lactis* NZ9000**							
ORF1	47,107	907	28.2	314	Toprim domain-containing protein, *Lactococcus lactis*	314/314 (100%)	WP_011997519.1
ORF2	1,579	2,748	34.2	389	RepB family plasmid replication initiator protein, *L. lactis*	389/389 (100%)	WP_058218799.1
ORF3	3,181	3,543	29.7	120	Hypothetical protein		
ORF4	3,863	3,594	23.7	89	Hypothetical protein		
ORF5	4,312	4,046	19.5	88	Hypothetical protein, *L. lactis*	88/88 (100%)	WP_058218801.1
ORF6	4,609	4,325	21.4	94	Hypothetical protein, *Lactococcus*	94/94 (100%)	WP_052206610.1
ORF7	4,878	6,920	30.3	680	MobA/MobL family protein, *Lactococcus hircilactis*	680/680 (99%)	WP_153497008.1
ORF8	7,109	7,348	33.3	79	Hypothetical protein		
ORF9	7,349	7,954	27.3	201	Hypothetical protein, *Lactococcus*	201/201 (100%)	WP_010890663.1
ORF10	7,967	8,311	42.9	114	Protein TrsB, *Lactococcus*	114/114 (100%)	WP_010890627.1
ORF11	8,332	8,679	25.8	115	Protein TrsC, *Lactococcus*	115/115 (100%)	WP_010890628.1
ORF12	8,663	9,316	29.1	217	Conjugal transfer protein, *Lactococcus*	204/217 (100%)	WP_153497006.1
ORF13	9,328	11,346	31.4	672	Protein TrsE, *Lactococcus*	672/672 (99%)	WP_058218807.1
ORF14	11,339	12,757	28.0	472	Conjugal transfer protein, *L. lactis*	472/472 (99%)	WP_195928101.1
ORF15	12,750	13,916	35.7	385	C40 family peptidase, *L. hircilactis*	385/385 (100%)	WP_153497003.1
ORF16	13,929	14,546	26.2	205	Hypothetical protein, *Lactococcus petauri*	205/205 (100%)	WP_165707211.1
ORF17	14,533	14,904	23.3	122	Hypothetical protein (Thioredoxin), *Lactococcus*	122/122 (100%)	WP_021166219.1
ORF18	14,901	15,359	29.9	152	Conjugal transfer protein TrsJ, *Lactococcus*	152/152 (100%)	WP_010890634.1
ORF19	15,356	16,987	31.2	543	Type IV secretory system conjugative DNA transfer family protein, *Lactococcus*	543/543 (100%)	WP_153497001.1
ORF20	17,002	17,397	27.0	131	Hypothetical protein		
ORF21	17,412	18,248	31.5	278	Conjugal transfer protein TrsL, *Lactococcus*	278/278 (100%)	WP_021166217.1
ORF22	18,264	20,441	28.1	725	DNA topoisomerase III, *L. petauri*	725/725 (99%)	WP_165707213.1
ORF23	20,444	20,653	29.6	69	Hypothetical protein, *Lactococcus*	69/69 (100%)	WP_010890684.1
ORF24	20,656	21,723	28.6	355	Hypothetical protein, *Lactococcus*	355/355 (99%)	WP_143459739.1
ORF25	22,971	22,252	28.4	239	Hypothetical protein	239/239 (100%)	ARW71132.1
ORF26	23,654	24,373	37.0	239	IS6-like element IS*1216* family transposase, *L. lactis*	239/239 (99%)	WP_060416618.1
ORF27	24,657	24,427	37.7	76	Hypothetical protein, *Staphylococcaceae*	76/76 (100%)	WP_011997529.1
ORF28	25,134	25,493	36.1	119	Helix-turn-helix domain-containing protein, Bacilli	119/119 (100%)	WP_002405438.1

ORF29	28,202	26,262	33.3	646	Tetracycline resistance ribosomal protection protein Tet(S), *Enterococcus faecium*	646/646 (100%)	ABB97394.1
ORF30	29,234	29,031	28.0	67	Conjugal transfer protein, *Staphylococcaceae*	67/67 (100%)	WP_017370349.1
ORF31	29,312	29,998	37.4	228	IS*6*-like element IS*1216* family transposase, *Enterococcus faecium*	228/228 (99%)	PQG28801.1
ORF32	30,200	30,021	28.3	59	rRNA adenine methyltransferase, *E. faecium*	59/59(93%)	WP_181046556.1
ORF33	30,882	30,145	33.1	245	23S rRNA (adenine(2058)-N(6))-methyltransferase Erm(B), Bacteria	245/245 (100%)	WP_001038795.1
ORF34	31,378	31,141	34.2	79	Peptide-binding protein, Bacteria	79/79 (99%)	WP_000635249.1
ORF35	31,607	32,293	39.1	228	Transposase, *L. lactis* subsp. *lactis* bv. *diacetylactis*	228/228 (99%)	AEK97256.1
ORF36	32,626	32,471	26.3	51	Hypothetical protein		
ORF37	33,109	32,933	26.9	58	Hypothetical protein		
ORF38	33,058	33,942	37.0	294	Virginiamycin B lyase, *Streptococcus gallolyticus*	294/294 (92%)	WP_039692849.1
ORF39	34,000	34,605	33.1	201	Vat family streptogramin A O-acetyltransferase, *S.gallolyticus*	201/215 (94%)	WP_039692847.1
ORF40	35,042	35,491	31.8	149	Hypothetical protein, *Alkalibaculum bacchi*	149/149 (92%)	WP_113921140.1
ΔORF41	35,504	35,701	33.3	65	Nucleotidyltransferase domain-containing protein, *Halanaerobiaceae bacterium*	55/284 (64%)	NLM97131.1
ΔORF42	36,054	37,100	32.1	348	Antirestriction protein ArdA, *Tissierella pigra*	339/521 (93%)	WP_195838003.1
ORF43	37,251	37,793	31.3	180	Tunicamycin resistance protein, *Clostridium* sp.	178/195 (88%)	QAA32198.1
ΔORF44	38,919	38,401	33.7	172	IS*110* family transposase, Clostridiales bacterium	128/387 (97%)	HCX61363.1
ORF45	39,430	40,022	39.4	197	Phosphotransferase, Bacilli	197/197 (88%)	WP_101031675.1
ORF46	40,121	40,972	37.9	283	Aminoglycoside 6-adenylyltransferase (ANT(6)-Ia), *Campylobacter jejuni*	283/302 (88%)	EDP8307115.1
ΔORF47	41,380	42,159	42.3	259	PTS sugar transporter subunit IIC, *Latilactobacillus curvatus*	249/430 (99%)	WP_148484706.1
ORF48	42,196	42,876	38.4	226	IS6 family transposase, *E. faecium*	226/226 (100%)	WP_060811670.1
ORF49	43,850	42,906	31.6	315	Hypothetical protein		
ORF50	44,081	44,425	29.9	114	Hypothetical protein		
ORF51	44,533	45,105	29.0	190	Recombinase family protein, *L. hircilactis*	190/190 (100%)	WP_153497015.1
ORF52	45,193	45,960	27.9	255	ParA family protein, *L. lactis*	255/255 (100%)	WP_011997516.1
ORF53	45,963	46,262	27.7	99	Hypothetical protein, *Lactococcus*	99/99 (100%)	WP_011997517.1
ORF54	46,330	46,695	28.6	121	Hypothetical protein, *L. hircilactis*	121/121 (100%)	WP_011997518.1
ORF55	46,710	47,090	30.4	126	Single-stranded DNA-binding protein, *Lactococcus*	126/126 (100%)	WP_010890681.1

[a]Including start and stop codons.

[b]Average G+C content of pTC1 (33.8%), pTC2 (34.3%), TC3 (32.6%), pTC4 (31.7%), pTC5 (30.6%), pTC6.1 (34.3%), pTC6.2 (39.5%), pTC7 (36.8%), and pERM1 (31.0%).

[c]aa, amino acids.

Table S2.- Primers and PCR conditions utilized in this study, as well as amplicon size expected of the tetracycline and erythromycin resistance genes targeted.

Primers	Sequence	Gene target	Annealing temperature (ºC)	Amplicon size (bp)	Reference
DI	GAYACICCIGGICAYRTIGAYTT	RPP*	50	1,083	82
DII	GCCCARWAIGGRTTIGGIGGIACYTC				
Tet-1	GATCGACCAGGCTGGCGTTG	RPP	50	1,300	83
Tet-2	GGCTGATTGGTTCTCCTGCG				
tetW	AAGCGGCAGTCACTTCCTTCC	*tet*(W)	50	1,200	84
Tet-2	GGCTGATTGGTTCTCCTGCG				
DI	GAYACICCIGGICAYRTIGAYTT	*tet*(M)	55	1,513	85
TetM-R	CACCGAGCAGGGATTTCTCCAC				
TetS-FWT1	ATCAAGATATTAAGGAC’	*tet*(S)	45	573	85
TetS-RVT2	TTCTCTATGTGGTAATC				
TetO-FW1	AATGAAGATTCCGACAATTT	*tet*(O)	45	781	85
TetO-RV1	CTCATGCGTTGTAGTATTCCA				
TetK-FW1	TTATGGTGGTTGTAGCTAGAAA	*tet*(K)	45	348	85
TetK-RV1	AAAGGGTTAGAAACTCTTGAAA				
TetL-FW3	GTMGTTGCGCGCTATATTCC	*tet*(L)	45	696	85
TetL-RV3	GTGAAMGRWAGCCCACCTAA				
erm(A)-1	TCTAAAAAGCATGTAAAAGAA	*ermA*	48	645	86
erm(A)-2	CTTCGATAGTTTATTAATATTAGT				
ermB-1	CATTTAACGACGAAACTGGC	*ermB*	50	639	86
ermB-2	GGAACATCTGTGGTATGGCG				
erm(C)-1	TCAAAACATAATATAGATAAA	*ermC*	43	642	86
erm(C)-2	GCTAATATTGTTTAAATCGTCAAT				
erm(F)-1	CGGGTCAGCACTTTACTATTG	*ermF*	50	466	87
erm(F)-2	GGACCTACCTCATAGACAAG				
mef(A)-1	ACCGATTCTATCAGCAAAG	*mefA*	43	940	88
mef(A)-2	GGACCTGCCATTGGTGTG				

*RPP, genes encoding ribosomal protection proteins.
